# Optimizing OCT acquisition parameters for assessments of vitreous haze for application in uveitis

**DOI:** 10.1038/s41598-018-20092-y

**Published:** 2018-01-26

**Authors:** G. Montesano, C. M. Way, G. Ometto, H. Ibrahim, P. R. Jones, R. Carmichael, X. Liu, T. Aslam, P. A. Keane, D. P. Crabb, A. K. Denniston

**Affiliations:** 1 City, University of London, Optometry and Visual Sciences, London, United Kingdom; 2 Academic Unit of Ophthalmology, Institute of Inflammation and Ageing, University of Birmingham, Birmingham, United Kingdom; 3 Department of Ophthalmology, University Hospitals Birmingham NHS Foundation Trust, Birmingham, United Kingdom; 4 NIHR Biomedical Research Centre at Moorfields Eye Hospital and UCL Institute of Ophthalmology, London, United Kingdom; 5 Manchester Royal Eye Hospital, Central Manchester University Hospitals NHS Foundation Trust, Manchester Academic Health Science Centre, Manchester, United Kingdom; 6 Faculty of Medical and Human Sciences, University of Manchester, Manchester, United Kingdom; 7 School of Built Environment, Heriot-Watt University, Edinburgh, United Kingdom

## Abstract

Detection and evaluation of inflammatory activity in uveitis is essential to the management of the condition, and yet continues to be largely dependent on subjective clinical measures. Optical coherence tomography (OCT) measurement of vitreous activity is an alternative to clinical vitreous haze scoring and has passed a number of early validation studies. In this study we aimed to evaluate the impact of ‘operator factors’ on the variability of the technique as part of the validation process, and to help evaluate its suitability for ‘real world’ use. Vitreous haze index was calculated as a ratio between the reflectivity of the vitreous and of the outer retina in each scan. Different scanning conditions were tested and their effect on the measurement is reported. Our results show that the ‘quantitative imaging’ technique of OCT-measured vitreous activity had good reliability in normal subjects under a range of ‘real world’ conditions, such as when the operator changes the averaging value. The technique was however vulnerable to highly inaccurate focussing or abnormal downward displacement of the image. OCT-based quantification of vitreous activity is a promising alternative to current subjective clinical estimates, with sufficient ‘tolerance’ to be used in routine clinical practice as well as clinical trials.

## Introduction

Uveitis is a group of diseases characterized by intraocular inflammation which collectively are a major cause of blindness worldwide^1–4^. One core objective in diagnosis and treatment is the correct identification and measurement of inflammatory activity^5,6^. This assessment has major impact both on routine clinical practice and on endpoint definition in clinical trials. Traditionally, the National Eye Institute (NEI) system for grading of vitreous haze has been the major disease activity endpoint for trials in posterior segment-involving uveitis, acknowledged by the United States Food and Drug Administration (FDA) and European Medicines Agency (EMA)^7,8^. However, the NEI system suffers from being (1) subjective, (2) noncontinuous, (3) poorly discriminatory at lower levels of inflammation, and (4) poorly sensitive in a clinical trial context^5,6,9^. A novel, automated method for the quantification of vitreous haze using optical coherence tomography (OCT) imaging was recently introduced^10^ thus providing objective measurement of vitreous inflammation. The method was based on a previously published study^11^, using a semi – automated implementation to correlate clinical vitreous haze scores in patients with uveitis and in healthy volunteers. The fully automated method was introduced to avoid the manual segmentation of OCT image sets by graders, a subjective and time-consuming step in the measuring process.

The new technique overcomes many of the well-known limitations of the NEI clinical score, and appears to be a major step forward in the drive towards sensitive objective endpoints for use in uveitis trials and to direct treatment decisions in routine clinical practice^12^. As part of its further validation it is important to determine what the potential limitations of this technique in the ‘real world’ – essentially what are the circumstances under which it would no longer be reliable. In general terms these can be considered as either ‘operator factors’ (dependent on how the technique is done) or ‘patient factors’ (intrinsic to the patient and their eye(s)).

In this report, we present a detailed analysis of the impact of ‘operator factors’ on the variability of the technique, with particular focus on the factors that can significantly affect the measure in healthy subjects where no inflammation is present. The experimental protocol was designed to test different scanning conditions using the Spectralis OCT (Heidelberg Engineering, Heidelberg, Germany). The analysis is aimed at the identification of the optimal acquisition settings that minimise the test-retest variability and changes in the measured value.

## Methods

### Scanning protocol

Fifteen volunteers with a refractive error within + 5 and −5 dioptres (D) were recruited. All subjects underwent a complete ophthalmic examination by an experienced clinician (AD) to confirm the absence of any pathologies. This protocol was approved from the NRES East Midlands Ethics Committee (Ref: 14/EM/1163). Written informed consent was gathered from all subjects. This protocol adhered to the tenets of the declaration of Helsinki.

Macular OCT scans centred on the foveal pit and spanning 20 degrees horizontally were acquired from the right eye of each subject using a spectral domain OCT device (Spectralis SD – OCT, Heidelberg Engineering, Heidelberg, Germany) with a 30-degree lens. An experienced operator used 10 different acquisition settings, each repeated 3 times, to acquire 30 raster scans (7 sections per scan) from each subject. Five Automated Real Time (ART) levels and five focus levels of the retina in the infrared (IR) fundus image were used in the acquisition protocol, as shown in Table 1. The ART level indicates the number of images that are averaged to produce the image of a single section. The positioning of the retina was set to the middle of the scan. This choice was forced by the final application of the proposed methodology, aimed at the measurement of the vitreous haze in patients with uveitis where macular oedema can be present. In fact, the presence of oedema forces the positioning to the middle of the scan in order to capture whole thickness of the swollen retina. As an additional comparison, one acquisition setting included the bottom positioning, ART 100 and in focus.

**Table 1:** Scanning protocol.

Vertical position of retina within the scan	ART Level	Focus
Middle	100	In focus
Middle	50	In focus
Middle	25	In focus
Middle	12	In focus
Middle	6	In focus
Middle	100	+5
Middle	100	+10
Middle	100	−5
Middle	100	−10
Bottom	100	In focus

Different setting combinations used during acquisition. Each scan with a specific setting has been repeated three times. ART = Automated Real Time.

Table 1 reports the different settings of the acquisition protocol in detail.

### Image analysis

**Figure 1:**
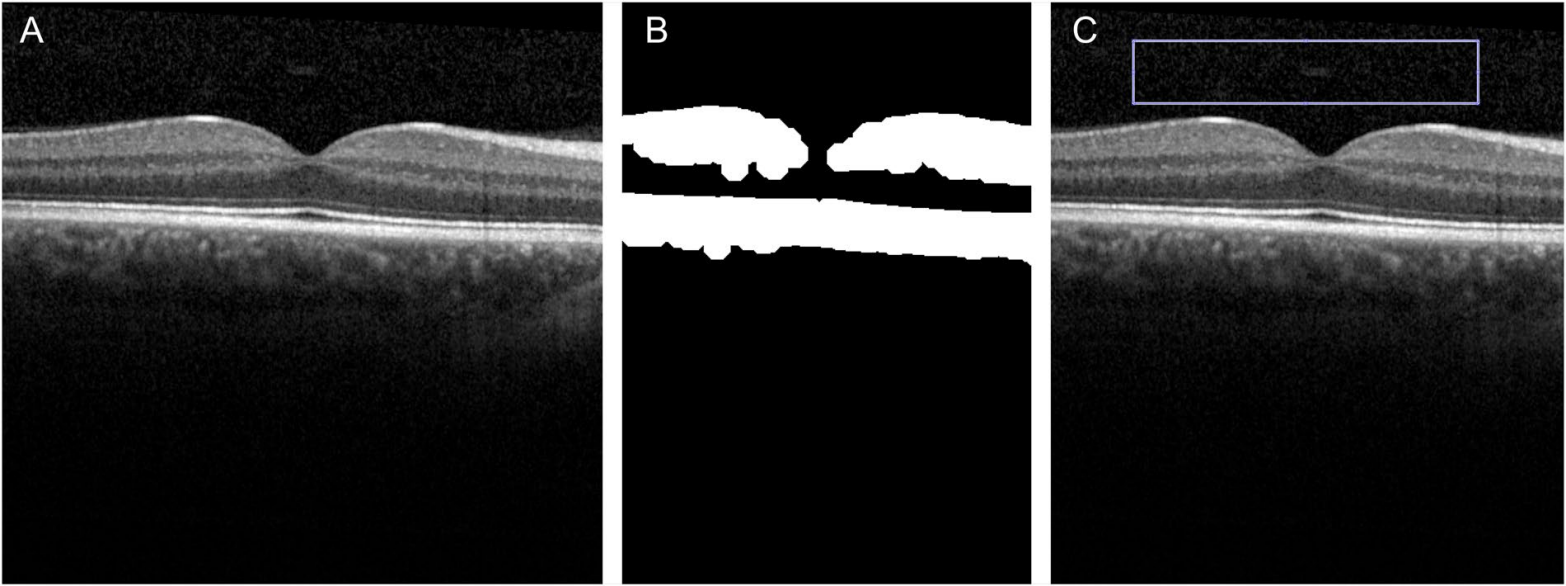
VITAN procedure. (**A**) Example original image. (**B**) Binary image of OCT scan automatically segmented to highlight retinal/RPE layers and cropped to isolate central areas. (**C**) Final automated area of capture overlaid onto original image for user approval.

To calculate the Vitreous/RPE-relative intensity (VRI), each image was analysed with the VITreous ANalysis (VITAN) software^10^, implemented in MATLAB (The MathWorks, Natick, MA, USA). Briefly, for each scan a morphological opening to segment the retina and RPE within the image was performed. Then, a vitreous patch was automatically generated based on this segmentation, excluding any retinal tissue (Fig. 1). The mean intensity of the vitreous patch and of the segmented RPE was measured and the ratio was calculated. The RPE intensity was used as a normalisation term, compensating for global reduction in the signal strength arising from diffused media opacities. The VITAN software then exported the VRI ratio, the vitreous mean intensity and the RPE mean intensity to a spreadsheet for analysis.

### Statistical analysis

Linear mixed models were used to assess the effect of different settings on the VRI. ART level and focus were analysed separately, with the ratio as the response variable. Observations consisted of the ratio calculated from each image of the scan. Clustering of sections within the same raster scan and of different repetitions within the same subject was addressed using nested random effects^13^. Due to the discrete nature of the settings, ART level and focus were used as factors rather than continuous variables. The same analysis was used to analyse separately the Vitreous and RPE intensities to calculate the effect of different acquisition parameters on these two values.

A similar approach was employed for the analyses of the variability of the measured ratio at three different levels: within the same raster scan, within subjects (intra-subject) and across subjects (inter-subjects). In this approach, the residuals of the measurement represented the observations. At each level, residuals were calculated as the difference (1) between each measurement and the mean of the seven sections in the raster scan, (2) between each mean of the raster scan and the mean of the three-repeated acquisitions and (3) between the mean of the acquisitions in the single subject and the mean of acquisitions across all subjects. Then, the squared residuals were used to model the variability of the measure at each level (within the raster scan, within subjects and across subjects) while changing the value of the parameter of interest (ART level or focus). Assuming normality of the residuals, the squared residuals follow a chi-squared distribution, which is a special case of the Gamma distribution. Therefore, generalized linear models with a Gamma distributed error and a logarithmic link function were used to model the effect of the different settings on squared residuals. The variability was reported as the square root of the estimate obtained from the model of squared residuals.

When a significant effect was detected, pairwise comparisons were performed between different settings and a multiple test correction with the Tukey method was applied.

When failure of the VITAN algorithm could not provide the measurement from at least 3 of the 7 scans or from at least 2 of the 3 repetitions, the raster scan or the repetition was discarded from the analysis for the variability.

All analyses were performed in R (R Foundation for Statistical Computing, Vienna, Austria) and MATLAB.

### Data availability statement

The datasets generated during and/or analysed during the current study are available from the corresponding author on reasonable request.

## Results

Twenty-one scans with the +10 D and 7 with +5 D settings could not be obtained due to difficulties in the acquisition. The VITAN algorithm failed to obtain the measurement in 46 out of 1575 theoretical scans from those with different ART level (3% failure rate) and in 504 out of 1575 with different focus (32% failure rate).

### Effect of ART level on VRI value

In our set of images, the ART level had minimal non-significant effect on the VRI value (overall p - value = 0.08, values are reported in Table 2), with slightly higher values with ART 100.

**Table 2:** Effect of ART on the VRI.

ART	Global mean ratio	Within scan variability	Intra - subject variability	Inter - subject variability
6	0.043	0.010	0.006	0.008
12	0.043	0.009	0.007	0.010
25	0.043	0.010	0.007	0.007
50	0.039	0.010	0.007	0.012
100	0.048	0.011	0.007	0.016*

*P < 0.05; **P < 0.01. Second column reports the estimated mean ratio value for different ART settings. No significant difference could be detected. Variability with different settings is reported as the square root of the estimate from the squared residuals model. The asterisk indicates the only significant difference (p < 0.05) that could be detected in pairwise comparisons between different settings (ART 100 showed and increased variability compared to the ART 6 and ART 25). ART = Automated Real Time.

### Effect of ART level on VRI variability

**Figure 2:**
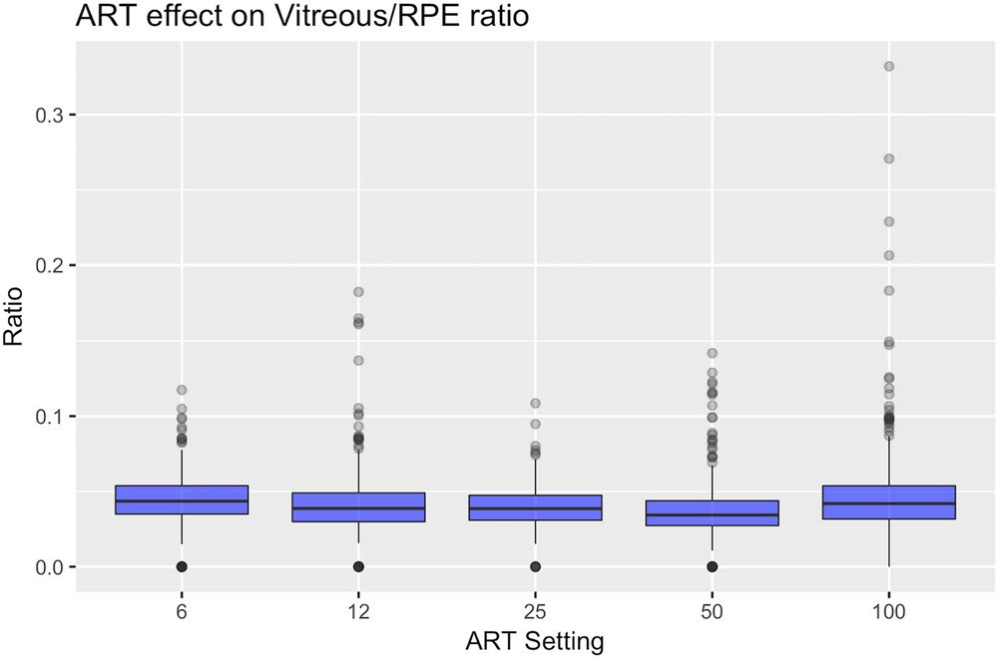
Effect of different ART settings on the VRI ratio. The box plot shows how different ART settings affect the mean VRI value and its variability. The ratio value did not show important variations, with slightly higher and more variable values with ART 100 (Refer to Table 2). The boxes extend from the 25^th^ to the 75^th^. Outliers (black dots) are points more distant than. The whiskers extend 1.5 times the interquartile range from the box limits. Points exceeding this limits are flagged as outliers (black dots). ART = Automated Real Time.

Modelling the squared residuals according to different ART levels revealed no significant effects on the variability of different sections within each raster scan (overall p-value = 0.308) and within different raster scans on the same subject (intra-subject variability, overall p-value = 0.869). A moderate effect could be found on the variability across subjects (inter-subject variability, overall p-value = 0.005). In pairwise comparisons, the ART 100 yielded higher variability compared to ART 6 (p = 0.032, 3.99-fold increase) and ART 25 (p = 0.004, 5.41-fold increase). Estimates from the model for variability are reported in Table 2. Figure 2 shows a graphical depiction of these results with a box plot graph.

### Effect of Focus on VRI value

In contrast with the analysis of the ART level, the analysis of the focus showed that this parameter had a major effect on the VRI (overall p-value < 0.001). All acquisition out of focus (referred to the retinal IR image) increased the VRI significantly (Minimum difference +Standard Error: 0.039 ± 0.008; p < 0.001), with larger increase using positive offsets (0.14 ± 0.008 increase for +5 D and 0.15 ± 0.01 for + 10 D). Results are reported in Table 3.

**Table 3:** Effect of focus on the VRI.

Focus	Global mean ratio	Within scan variability	Intra - subject variability	Inter - subject variability
−10 D	0.162**	0.019**	0.015**	0.036
−5 D	0.088**	0.013	0.012*	0.029
**In focus**	**0.048**	**0.011**	**0.007**	**0.023**
+5 D	0.192**	0.032**	0.022**	0.040*
+10 D	0.200**	0.027**	0.033**	0.036

*P < 0.05; **P < 0.01. The second column reports the estimated mean ratio value for different focus settings. All p – values have been calculated comparing each other level to the “In focus” condition (in bold). Variability with different settings is reported as the square root of the estimate from the squared residuals model. The asterisks indicate significant differences according to the legend at the bottom of the table.

### Effect of Focus on VRI variability

**Figure 3:**
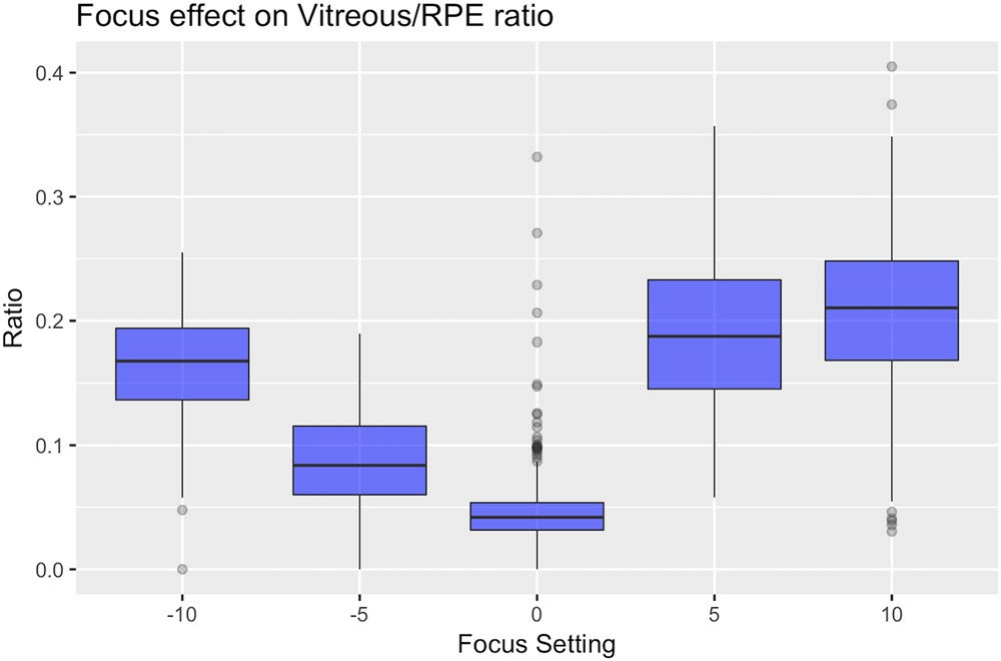
Effect of different focus settings on the VRI. The box plot shows how different focusing condition increase the mean VRI value and its variability compared to scans focused on the retina (denoted as 0 in the graph). The boxes extend from the 25^th^ to the 75^th^. Outliers (black dots) are points more distant than. The whiskers extend 1.5 times the interquartile range from the box limits. Points exceeding this limits are flagged as outliers (black dots).

As shown in Fig. 3, Focus significantly affected variability at all levels (within scans, within subjects and across subjects). All settings that deviated from the optimal retinal focus caused a significant increase in within scan variability (all p < 0.001) except for the −5 D condition (p = 0.08). A significant increase in the within subject variability was observed in any focus offsets (all p < 0.018), while only the +5 D caused a significant increase in variability across subjects (p = 0.038). Among all settings, positive offsets caused the largest increase in variability compared to the in focus condition. Values are reported in Table 3.

### Effect of vertical positioning on the VRI

When compared to standard (middle) positioning within the z-plane, relatively inferior positioning of the retinal image within the acquisition frame also significantly increased the VRI value (Estimated difference ± Standard Error: 0.114 ± 0.007; p < 0.001) and the variability at all levels (all p-values < 0.01).

### Differential contribution of Vitreous and RPE intensity on the VRI

The individual variation of the two measured components of the ratio (Vitreous intensity and the RPE intensity) is reported in Table 4 for the acquisition parameters that significantly affected the measurement (i.e. focus and positioning). Variation is reported with the absolute difference and the percentage relative to the reference levels of each setting: ‘in focus’ for the focus and ‘middle’ for the positioning. For different settings of the focus, the major contribution to the variation in the ratio was due to changes in the vitreous intensity, particularly for positive offsets. Conversely, the increase in vitreous intensity observed with the bottom positioning was caused by both the increase of Vitreous intensity (the numerator of the ratio) and the reduction of RPE intensity (the denominator). Independently of their magnitude, all variations were statistically significant (p < 0.05).

**Table 4:** Different contributions to the VRI.

		Ratio		Vitreous		RPE	
		Difference	Perc. Change (%)	Difference	Perc. Change (%)	Difference	Perc. Change (%)
Focus	**−10 D**	0.12	239.78	23.948	262.79	16.91	9.22
	−**5 D**	0.04	82.56	8.955	98.27	21.51	11.72
	**5 D**	0.14	297.45	27.677	303.71	6.16	3.36
	**10 D**	0.15	315.49	31.065	340.89	14.26	7.77
Position	**Bottom**	0.11	70.30	19.051	67.64	−14.27	−8.43

The table reports the differences in VRI ratio values (column 3), vitreous intensity (column 5) and RPE intensity (column 7) compared to their respective reference levels (the “In focus” condition for focus and the “Middle” location for position). Columns 4, 6 and 8 report the same changes as percentage increase (or decrease if negative) from the reference level.

## Discussion

Our previous work showed that the measurement of the VRI from OCT scans is correlated with the clinical score for vitreous haze in uveitis patients^11,14^ and that it could be partially automated^10^ and that it was highly sensitive to detecting treatment responses^15^. However, in order to assess whether the VRI can be used in routine clinical practice to detect pathological vitreous haze, it is crucial to study how this measurement can vary using different acquisition settings. This report investigates the extent to which ‘operator factors’ such as the effect of image averaging (ART level), defocussing and retinal positioning might impact the reliability of the technique. This is particularly important when considering a technique that is intended for use in everyday clinics and not just in the more controlled environment of a clinical trial.

The ART level is used to improve the quality of the images via averaging by increasing signal to noise ratio^16^. The analysis showed a mild, non-significant effect of image averaging (p = 0.078) on the ratio measurement (Table 2), with slightly higher values obtained using ART 100. The maximum difference obtained between estimated values was 0.0093. Such a difference is well below the observed increase with vitritis, reported in our previous retrospective analysis (difference in medians, Vitritis – Healthy group = 0.0733)^11^. ART 100 showed a higher inter-subject variability (overall p-value = 0.005), but only pairwise comparisons with the ART 6 and ART 25 were significant. No significant effect could be detected for the within-scan and intra-subject variability. These results are compatible with the fact that image averaging should make the vitreous intensity converge toward a mean value, with no major impact on the ratio value. However, averaging can occasionally smooth out sharp features^17^ and change the textural properties of the vitreous. This effect could have an impact on the analysis of images from patients with uveitis by reducing the discriminability between diffuse haze and residual, small clumps of the vitreous in the absence of an active inflammatory processes. From a clinical perspective, it is important to notice that no significant differences could be detected across scans with lower ART values. In the clinical evaluation of macular oedema, a raster scan with the default ART (9) is acquired as a trade-off between image quality and acquisition speed. Results show that VRI can be safely calculated without changing the standard acquisition setting in clinical routine. This result could allow a retrospective application of the measurement, even in sets of OCT images that have not been acquired for this specific purpose.

Changes in the focus had a high impact on the VRI. Different OCT imaging of the vitreous can be obtained with different focusing^18,19^. Although vitreous details can be better imaged with anterior focusing (positive offsets), a more accurate resolution of vitreous structures can falsely increase the VRI and fail to highlight the diffuse haze due to inflammation. This was well reflected by the increase in vitreous intensity observed when changing the focus and was more prominent when using positive offsets (Table 4). Measurement variability was also greatly affected by the focus and particularly by positive offsets. Increased variability across different sections of the same scan (within scan variability) can be explained by the presence of vitreous structures, varying in density as the scan location moves from the inferior to the superior part of the macular cube. This could have also been the cause of the overall greater variability in the ratio value on scan repetitions (possibly due to slight shifts in the location of the acquisition pattern each time) and in the inter-subject variability where only the +5 D offset was significantly different (possibly due to a better focusing on the vitreous and thus more affected by inter individual changes in the vitreous structure). Increased variability and vitreous values resulting from posterior focusing might be related to an increase in noise and a relative decrease in the signal to noise ratio, with a worse resolution of the RPE and of the vitreous signal.

Finally, the position of the retinal section within the scan also affected the ratio significantly, increasing the ratio value and the variability of the measure when displaced to the bottom. This change with the bottom positioning might constitute a limitation when imaging patients with important macular oedema, as the RPE is forcefully moved downward in the scan to accommodate the entire retinal thickness in the scan. As shown in Table 4, this increase might be due to the combined effect in the reduction of the RPE intensity with the bottom displacement (possibly a consequence of the known fading effect at the edges of the acquisition window) and to the increase of the vitreous intensity (due to an increase in the noise in the analysed vitreous patch).

This study forms part of the ongoing validation process for a ‘quantitative imaging’ approach to vitreous haze using OCT. We recognise that one of the limitations of this study is that it did not deal with all possible reliability factors but deliberately focused on ‘operator factors’ rather than ‘patient factors’. ‘Patient factors’ include the effect of media opacities and ocular surface issues. Media opacities and tear film inhomogeneity are known factors affecting the quality and the signal to noise ratio in OCT scans^16,20^, and might falsely increase the measured vitreous haze. An in-depth analysis of these aspects will be possible with a large cohort of normal subjects with a wide age range. Given its focus on ‘operator factors’ this study was undertaken on healthy controls, and so, unlike most of our previous studies, did not allow a discrimination analysis to investigate the ability of detecting vitritis in uveitis patients. Further investigation of variability and discriminative power of the method will be undertaken as part of a major validation study (OCTAVE) which will also evaluate the impact of increasing the volume sampled through alternative OCT acquisition protocols (eg wide-angle OCT and extra-macular OCT). Lastly, most OCT devices present Gamma-transformed images to increase the contrast of the retinal layers. However, this might not be the optimal condition for vitreous analysis. Measurements obtained from raw, unprocessed data might be more suitable in order to precisely quantify the signal intensity.

In conclusion, this study in healthy subjects suggests that the OCT-based VRI ratio is reasonably tolerant of ‘operator factors’ and would remain reliable if transferred from a clinical trial setting to the ‘real world’. Additional validation studies are ongoing to evaluate the impact of ‘patient factors’ on reliability, and to assess repeatability and discrimination in a prospective cohort of patients with uveitis as part of the OCTAVE study.

## References

[1] Durrani OM, Meads CA, Murray PI (2004). Uveitis: a potentially blinding disease. Ophthalmologica. Journal international d’ophtalmologie. International journal of ophthalmology. Zeitschrift fur Augenheilkunde.

[2] Wakefield D, Chang JH (2005). Epidemiology of uveitis. International ophthalmology clinics.

[3] Rothova A, Suttorp-van Schulten MS, Frits Treffers W, Kijlstra A (1996). Causes and frequency of blindness in patients with intraocular inflammatory disease. The British journal of ophthalmology.

[4] Nussenblatt RB (1990). The natural history of uveitis. International ophthalmology.

[5] Lin P, Suhler EB, Rosenbaum JT (2014). The future of uveitis treatment. Ophthalmology.

[6] Denniston AK, Dick AD (2013). Systemic therapies for inflammatory eye disease: past, present and future. BMC ophthalmology.

[7] Nussenblatt RB, Palestine AG, Chan CC, Roberge F (1985). Standardization of vitreal inflammatory activity in intermediate and posterior uveitis. Ophthalmology.

[8] Jabs DA, Nussenblatt RB, Rosenbaum JT, Standardization of Uveitis Nomenclature Working, G. (2005). Standardization of uveitis nomenclature for reporting clinical data. Results of the First International Workshop. American journal of ophthalmology.

[9] Madow B, Galor A, Feuer WJ, Altaweel MM, Davis JL (2011). Validation of a photographic vitreous haze grading technique for clinical trials in uveitis. American journal of ophthalmology.

[10] Keane PA (2015). *Automated Analysis of Vitreous Inflammation* Using Spectral-Domain Optical Coherence Tomography. Translational vision science & technology.

[11] Keane PA (2014). Objective measurement of vitreous inflammation using optical coherence tomography. Ophthalmology.

[12] Denniston AK, Keane PA, Srivastava SK (2017). Biomarkers and Surrogate Endpoints in Uveitis: The Impact of Quantitative Imaging. Investigative ophthalmology & visual science.

[13] Bates, D., Mächler, M., Bolker, B. & Walker, S. Fitting Linear Mixed-Effects Models Usinglme4. *Journal of Statistical Software* **67**, 10.18637/jss.v067.i01 (2015).

[14] Zarranz-Ventura J (2016). Evaluation of Objective Vitritis Grading Method Using Optical Coherence Tomography: Influence of Phakic Status and Previous Vitrectomy. American journal of ophthalmology.

[15] Sreekantam S (2017). Quantitative analysis of vitreous inflammation using optical coherence tomography in patients receiving sub-Tenon’s triamcinolone acetonide for uveitic cystoid macular oedema. The British journal of ophthalmology.

[16] Podkowinski D (2017). Impact of B-Scan Averaging on Spectralis Optical Coherence Tomography Image Quality before and after CataractSurgery. Journal of ophthalmology.

[17] Gelfand JM, Nolan R, Schwartz DM, Graves J, Green AJ (2012). Microcystic macular oedema in multiple sclerosis is associated with disease severity. Brain: a journal of neurology.

[18] Takahashi A, Nagaoka T, Yoshida A (2016). Enhanced vitreous imaging optical coherence tomography in primary macular holes. International ophthalmology.

[19] Pang CE, Freund KB, Engelbert M (2014). Enhanced vitreous imaging technique with spectral-domain optical coherence tomography for evaluation of posterior vitreous detachment. JAMA ophthalmology.

[20] Stein DM (2006). Effect of corneal drying on optical coherence tomography. Ophthalmology.

